# Wide excision and latissimus dorsi flap reconstruction for advanced axillary hidradenitis suppurativa: a case series

**DOI:** 10.1080/23320885.2026.2639268

**Published:** 2026-02-28

**Authors:** Frank Andrés Álvarez Vásquez, Iván Enrique Rodríguez Mantilla, Liceth Lorena Patarroyo Villalobos, Nicol Daniella Cala Gómez, Jorge Luis Corcho Acosta, Juan Pablo Zaraza Duarte

**Affiliations:** a Physician, Universidad del Rosario, Epidemiologist, Universidad Autónoma de Bucaramanga, Colombia; b Plastic Surgeon, Fundación Universitaria de Ciencias de la Salud. Fellowship in Advanced Oncologic Plastic Surgery, Reconstructive Microsurgery, and Lymphedema Surgery, Taichung, Taiwan; c Plastic Surgeon, Pontificia Universidad Javeriana. Fellowship in Microsurgery and Oncologic Plastic Surgery, Colombia; d Plastic Surgeon, Universidad Nacional de Colombia, Colombia; e Physician, Universidad del Rosario, Colombia

**Keywords:** Hidradenitis suppurativa, reconstructive surgery, Latissimus dorsi flap, perforator flaps

## Abstract

Hidradenitis suppurativa (HS) is a chronic inflammatory disorder of the pilosebaceous unit characterized by recurrent nodules, tunnels, scarring, and functional impairment, most commonly affecting intertriginous regions. Surgical excision addresses irreversible tissue damage, while medical therapy controls the underlying inflammatory process; both strategies should be considered complementary. Following wide excision, secondary intention healing remains a valid option for axillary defects; however, flap reconstruction may be electively chosen in selected patients to shorten healing time and reduce wound care burden based on patient preference and surgeon experience. This article presents a case series of three patients with Hurley stage III axillary HS who underwent wide excision followed by reconstruction with latissimus dorsi musculocutaneous flaps. Operative details, perioperative management, postoperative outcomes, and follow-up are reported. All patients achieved durable coverage, preserved shoulder range of motion, high aesthetic satisfaction, and no clinical recurrences during follow-up.

## Introduction

Hidradenitis suppurativa (HS), also known as inverse acne, is a chronic, relapsing autoinflammatory disorder of the pilosebaceous unit [1]. Clinically, it is characterized by recurrent abscesses, tunnels scarring, and fibrotic contractures, predominantly affecting intertriginous skin such as the axillary, inguinal, perineal, inframammary, and intergluteal areas [1].

HS most commonly manifests between the second and fourth decades of life, with a marked decline in incidence after the age of 55. In the United States, the age- and sex-adjusted incidence is approximately 6 cases per 100,000 individuals per year, with a substantially higher prevalence in women [2]. In Colombia, the estimated prevalence is 7.4 per 100,000 inhabitants; however, epidemiologic data across Latin America remain limited and likely underestimated due to diagnostic delays and under-recognition [2].

Multiple risk factors have been associated with increased disease susceptibility and severity, including female sex, a first-degree family history, obesity, smoking, metabolic syndrome, hormonal dysregulation, and aberrant immune responses [1–3]. HS demonstrates a mixed inflammatory infiltrate centered around the hair follicle, with heightened activity of neutrophils, monocytes, mast cells, and elevated levels of proinflammatory cytokines such as IL-1, IL-10, IL-17, and TNF-α [2,3].

Several clinical instruments have been developed to quantify disease activity and monitor therapeutic response, including the Modified Sartorius Score (mSS), Hidradenitis Suppurativa Physician Global Assessment (HS-PGA), Numeric Rating Scale (NRS), Hidradenitis Suppurativa Clinical Response (HiSCR), and the International Hidradenitis Suppurativa Severity Score System (IHS4) [1–4]. Nevertheless, the Hurley staging system remains the most widely used classification in routine practice due to its simplicity and practicality. Hurley stage I consists of isolated abscesses or inflammatory nodules without scarring or sinus tracts; stage II is characterized by recurrent abscesses with single or multiple tunnels and scarring; and stage III represents diffuse or near-diffuse involvement with multiple interconnected tunnels and deeply located abscesses, often resulting in functional and aesthetic impairment [4,5].

Management strategies range from topical and systemic medical therapy to surgical intervention depending on disease severity, anatomical involvement, and patient-specific factors. Traditional medical therapy includes topical clindamycin, systemic antibiotics, intralesional corticosteroids, immunosuppressants, and first-generation biologic agents such as adalimumab and infliximab [3–5]. However, recent advances in immunotherapy have significantly expanded the therapeutic landscape [6,7]. In 2025, the IL-17 inhibitors bimekizumab and secukinumab were approved for HS, demonstrating clinically meaningful reductions in inflammatory lesions and improvements in quality of life, including in patients who previously failed adalimumab therapy [6–8].

Additionally, JAK inhibitors have emerged as promising agents, with increasing evidence highlighting their role in modulating key inflammatory pathways involved in HS pathogenesis [7,8].

Despite these advances, patients with Hurley stage III disease often exhibit limited or incomplete response to medical therapy alone and frequently require surgical management for definitive disease control. Wide local excision remains the cornerstone of treatment for advanced axillary HS, enabling complete removal of affected tissue and reducing recurrence.

**Figure 1.**
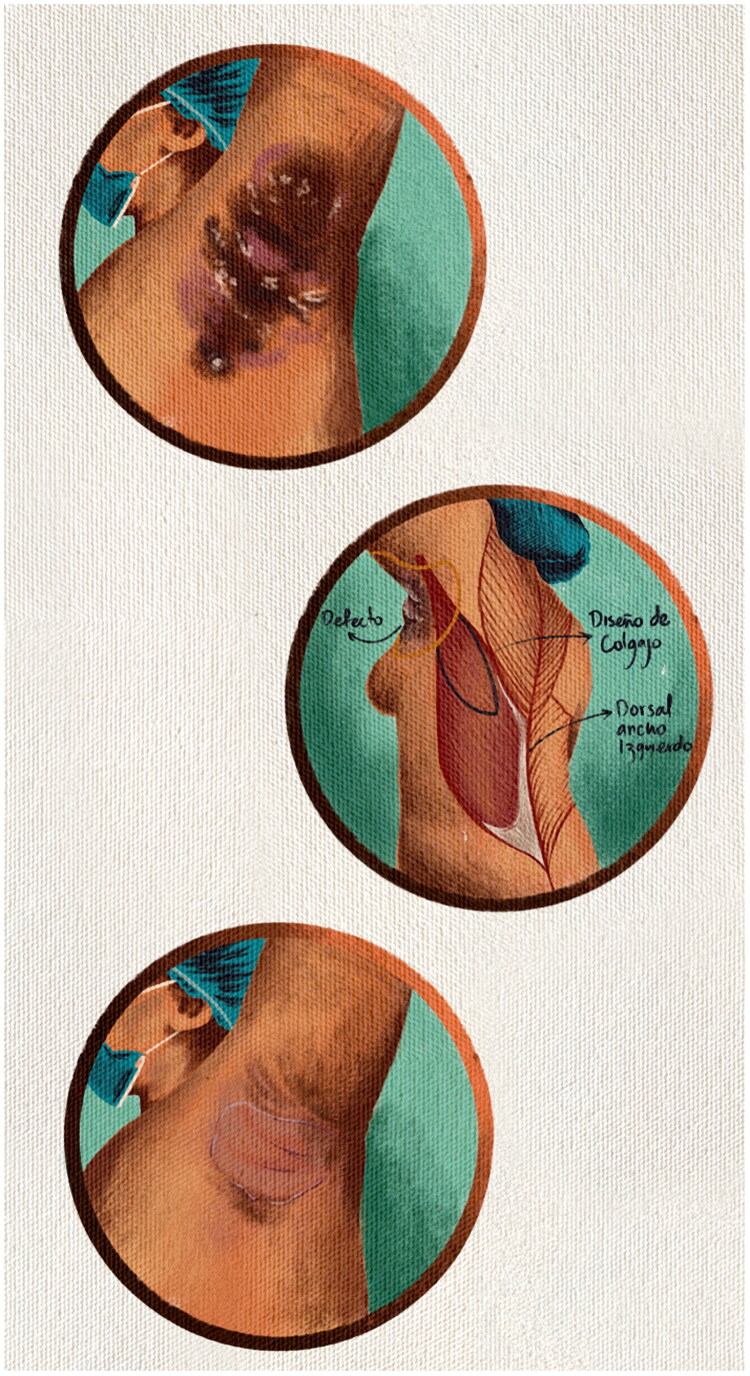
Clinical presentation and surgical planning in axillary hidradenitis suppurativa. Preoperative view demonstrating nodules, interconnected tunnels, and scarring in the axillary region consistent with Hurley stage III hidradenitis suppurativa. Markings indicate the planned area of wide excision and the design of the latissimus dorsi musculocutaneous flap for defect closure. Early postoperative outpatient follow-up demonstrates a viable flap without evidence of ischemia or early recurrence.

The objective of this study is to contribute to the reconstructive literature by presenting a case series of patients with severe axillary HS who underwent wide excision followed by reconstruction using latissimus dorsi musculocutaneous flaps, achieving favorable functional and aesthetic outcomes (Figure 1).

## Methods

A retrospective descriptive case series was conducted including three female patients aged 30–35 years diagnosed with Hurley stage III axillary HS, treated at a tertiary referral center in Bogotá, Colombia, between 2022 and 2025. All patients were receiving ongoing systemic medical therapy, including biologic agents, which was continued throughout the preoperative, perioperative, and postoperative periods in accordance with current HS management recommendations [6–9] (Table 1).

**Table 1.** Patient characteristics, extent of excision, operative time, hospitalization, postoperative pain control, complications, donor-site outcomes, and follow-up in three patients with Hurley stage III axillary hidradenitis suppurativa.

Patient	Age	Sex	Axxila	Hurley stage	Lesion size (cm)	Lesion characteristics	Post- excision defect size (cm)	Surgical excision	Reconstruction	Operative time	Anesthesia	Hospital stay	Drains	Drain removal postoperative day (pod)	Postoperative positioning	Complications	Donor-site morbidity	Recurrence	Functional outcome	Aesthetic outcome / patient satisfaction
1	32	F	Left	lll	15 × 8	Multiple inflammatory nodules, interconnected sinus tracts, hypertrophic scarring	19 × 10	Wide excision to healthy margins	Latissimus dorsi musculocutaneous flap	90 min	General	9 days	Axillary + donor site	12	Shoulder extension; lateral decubitus	None	None	None	Full Range of Motion (ROM) preserved	Satisfactory contour; high satisfaction
2	30	F	Right		13 × 7	Extensive fibrotic tissue with active draining sinuses	16 × 10	Wide excision to healthy margins				7 days		14						Good symmetry; high satisfaction
3	34	F	Left		9 × 6	Chronic inflammatory plaques with scarring and sinus tracts extending to lateral thoracic region	10 × 10	Wide excision to healthy margins				8 days		15						Symmetric axillary contour; high satisfaction

## Surgical technique

In all three cases, a wide excision of the affected tissue was performed under general anesthesia. To optimize the intraoperative identification of tunnels, methylene blue was injected subcutaneously, allowing for precise delineation of the diseased margins. Excisions were carried out down to the fascia, resulting in extensive axillary defects. All specimens were submitted for histopathological evaluation to confirm the diagnosis and rule out associated malignancies.

Following assessment of the resulting defects, three latissimus dorsi (LD) musculocutaneous flaps were designed on the ipsilateral dorsal region. Preoperative markings incorporated anthropometric references and anatomical landmarks, centered on the axis of the thoracodorsal artery. Handheld Doppler ultrasonography was employed to map the vascular course and confirm inclusion of the pedicle within the skin island. A transcutaneous incision was performed, followed by flap dissection in a lateral-to-medial direction, preserving the medial boundary adjacent to the serratus anterior muscle.

**Figure 2.**
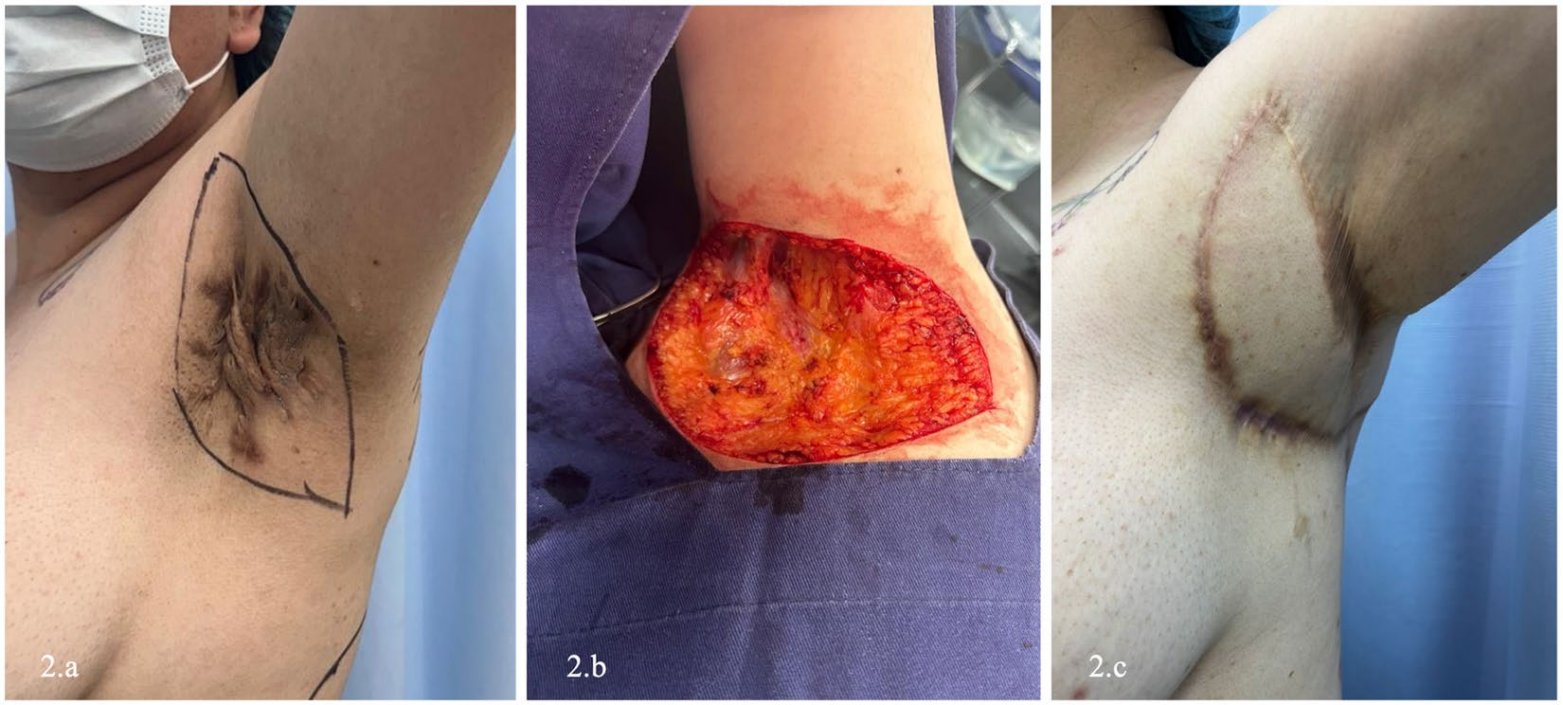
Wide excision and flap reconstruction of left axillary hidradenitis suppurativa. (a) Preoperative appearance of left axillary Hurley stage III disease with nodules, tunnels, abscess formation, and extensive cicatricial changes. (b) Axillary defect following wide excision, confined to the skin and subcutaneous tissue. (c) Immediate postoperative view after reconstruction with a latissimus dorsi musculocutaneous flap. A closed-suction drain was placed in the donor site. (d) Early postoperative follow-up demonstrating stable flap perfusion and intact wound closure.

**Figure 3.**
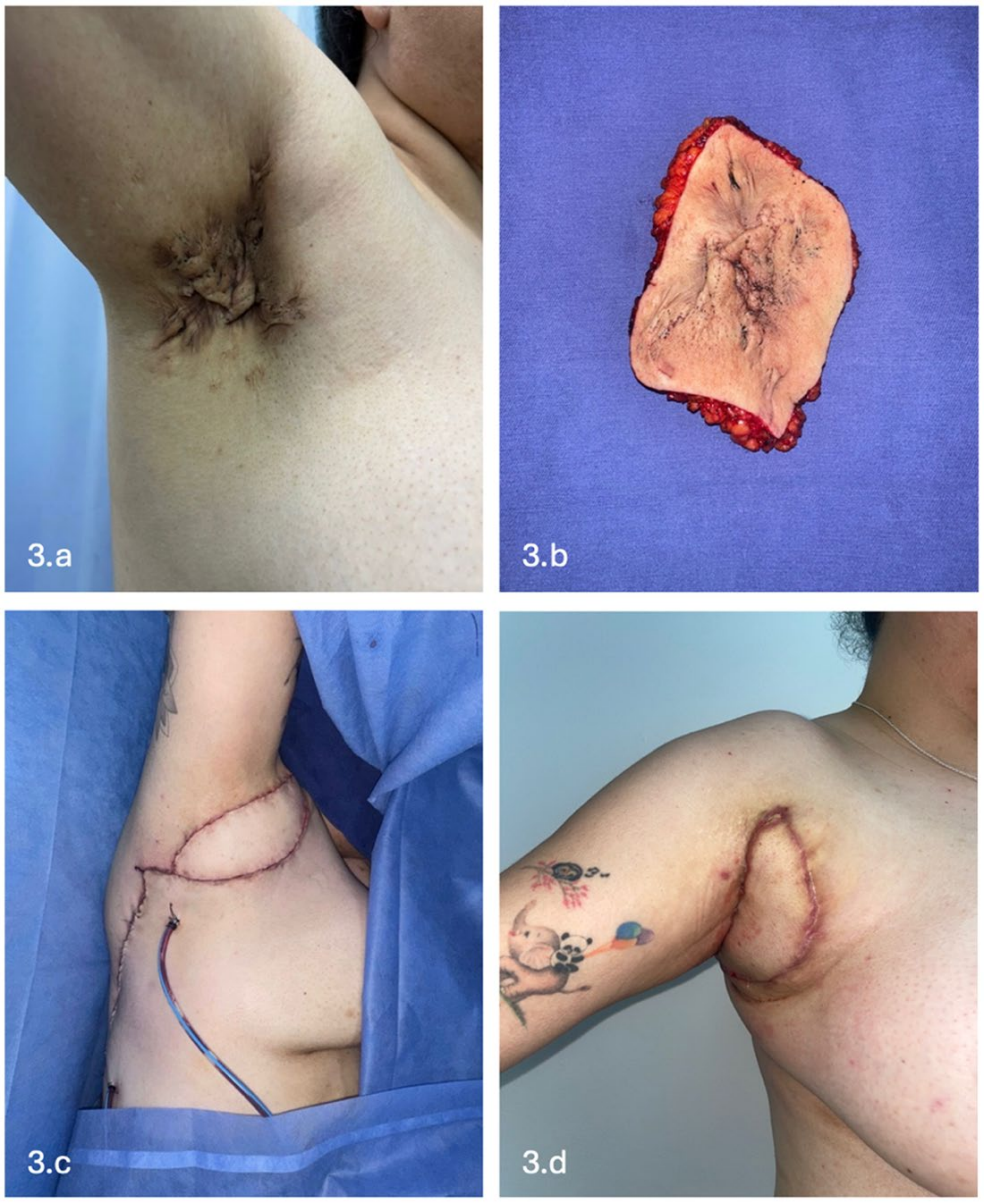
Right axillary Hurley stage III hidradenitis suppurativa treated with excision and flap reconstruction. (a) Preoperative image showing extensive nodules, tunnels, and scarring involving the right axillary region with extension to the adjacent lateral thoracic skin. (b) Resected specimen following wide excision. (c) Intraoperative view of the latissimus dorsi musculocutaneous flap with a well-perfused skin paddle prior to inset. (d) Early outpatient follow-up demonstrating stable defect coverage without wound dehiscence or flap compromise.

Dissection proceeded cephalad until identification of the bifurcation of the thoracodorsal vessels into the LD and serratus anterior branches (Figure 2). Utilizing microsurgical technique, meticulous submuscular dissection was performed to isolate and preserve the thoracodorsal neurovascular bundle. Once the pedicle was skeletonized and the muscle fully mobilized, the flap was rotated 180 degrees to provide tension-free coverage of the axillary vault. Flap perfusion was clinically confirmed after rotation (Figure 3).

**Figure 4.**
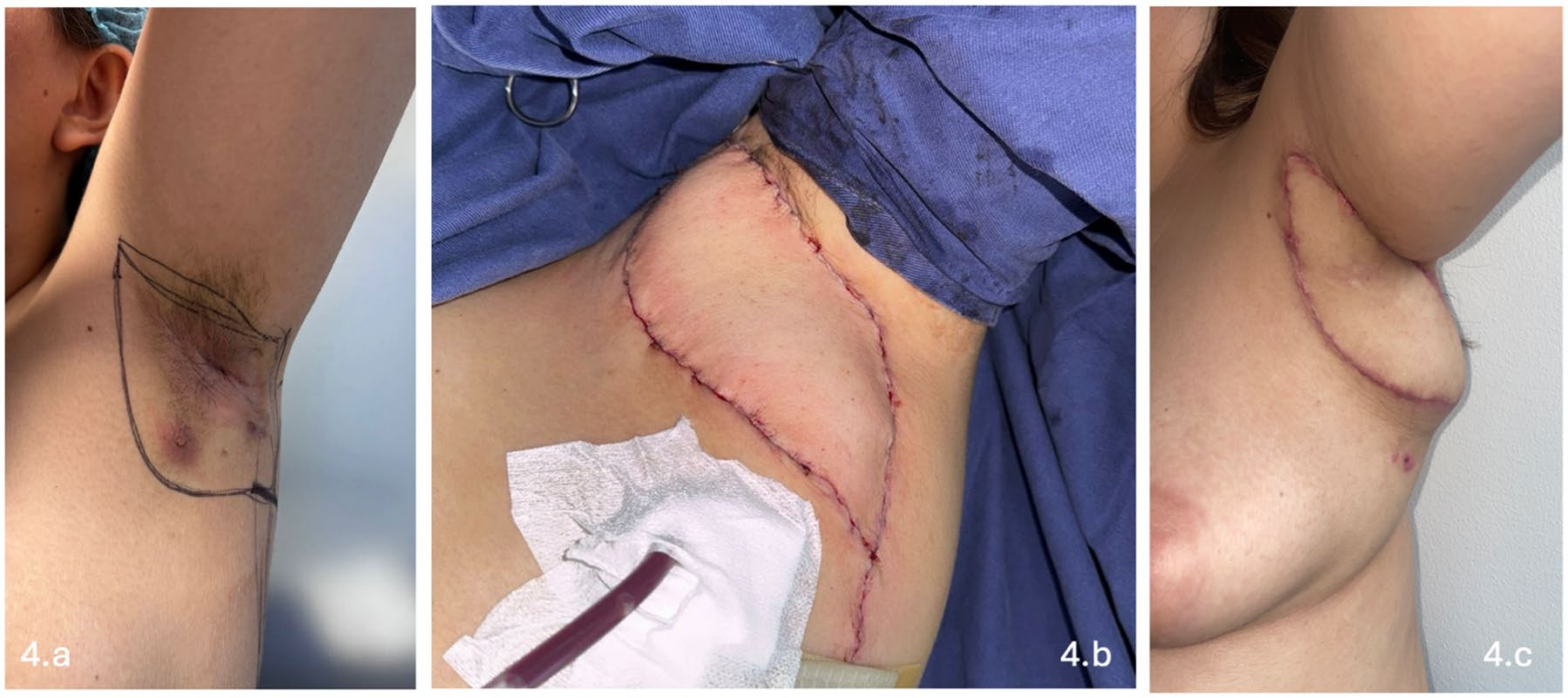
Left axillary hidradenitis suppurativa reconstructed with latissimus dorsi musculocutaneous flap. (a) Preoperative view of Hurley stage III disease involving the left axillary region, characterized by nodules, tunnels, and scarring. (b) Intraoperative view of the harvested latissimus dorsi musculocutaneous flap with adequate perfusion. (c) Postoperative outpatient follow-up demonstrating flap viability and preservation of axillary contour.

The muscle component was anchored to the deep tissues of the axillary fossa to obliterate dead space and minimize the risk of seroma formation. Two closed-suction drains were placed at the axillary recipient site, and additional closed-suction drains were placed at the donor site in all patients. Layered closure was performed with absorbable sutures for the deep and subcutaneous layers, and skin closure was completed using absorbable monofilament sutures (Monocryl^®^, poliglecaprone 25). In all three cases, the donor site was managed with primary closure, achieved through wide undermining to ensure a tension-free repair (Figure 4).

## Perioperative management and follow-up

All procedures were performed under general anesthesia, with a mean operative time of approximately 90 min (range, 85–95 min). Estimated intraoperative blood loss was minimal in all cases and did not require blood transfusion. No intraoperative complications were recorded.

Postoperative pain was managed using a multimodal analgesic regimen, including scheduled acetaminophen, nonsteroidal anti-inflammatory drugs, and opioid analgesics administered on an as-needed basis, according to institutional protocols and individual patient requirements.

Patients remained hospitalized for a mean duration of 8 days (range, 7–9 days). The primary indications for inpatient management included close flap monitoring, optimization of postoperative pain control, and comprehensive patient education regarding wound care, activity restrictions, and postoperative positioning.

Postoperatively, patients were instructed to maintain the surgical wounds dry at all times, avoid any direct pressure over the flap, and keep the operated shoulder in extension. In addition, patients were advised to remain in lateral decubitus positioning during the immediate postoperative period in order to minimize compression of both the flap and the donor site and to optimize flap perfusion.

Surgical drains were removed during outpatient follow-up between postoperative days 12 and 15, once drainage output was deemed minimal and clinically acceptable. Follow-up was conducted on an outpatient basis, with scheduled weekly evaluations during the first postoperative month, followed by periodic assessments thereafter, according to standard institutional follow-up protocols.

Although healing by secondary intention was discussed as a valid management option for all patients, flap reconstruction was ultimately selected based on patient preference for a shorter overall healing time, reduced wound care requirements, and an earlier return to daily activities. This decision was also supported by surgeon experience and preference for flap-based reconstruction in this anatomical region.

No cases of partial or total flap loss, wound dehiscence, surgical-site infection, hematoma, seroma, or donor-site morbidity were observed throughout the postoperative course. Postoperative positioning protocols were followed in all patients, and no patients demonstrated any limitation in final shoulder mobility at last follow-up.

No clinical recurrences were identified during the follow-up period. All patients achieved full shoulder range of motion and reported high levels of satisfaction with both functional and aesthetic outcomes. In addition, all patients expressed high satisfaction with overall recovery, particularly with respect to reduced wound care burden and an earlier return to daily activities when compared with their expectations for healing by secondary intention.

## Discussion

Surgical excision remains a central component of management for patients with advanced-stage hidradenitis suppurativa (HS) or those with extensive lesions within a defined anatomical region [10]. Contemporary recommendations emphasize describing procedures based on anatomical extent rather than fixed margin measurements [10,11]. Terms such as lesional excision (removal of discrete affected tissue) or regional excision (removal of all disease within a topographically defined area) are preferred, with modifiers like partial or complete to clarify the scope of resection [12,13]. This standardized nomenclature facilitates comparison across studies and avoids ambiguity in reporting outcomes [10]. HS lesions typically remain within the dermis and subcutaneous fat, with tunnels forming within these layers; deeper tissue involvement is uncommon [14].

The choice to reconstruct versus allow healing by secondary intention should be individualized [14,15]. Secondary intention healing remains a valid and effective approach for all axillary defects, regardless of size, although larger wounds require longer healing times. Advanced reconstructive techniques, including local, regional, or musculocutaneous flaps, are generally employed to shorten recovery time, reduce wound care demands, and improve early aesthetic and functional outcomes, particularly in patients with extensive defects who prefer a faster return to daily activities. Decisions should also account for patient preference, comorbidities, defect location, and surgeon experience [10,11].

**Figure 5.**
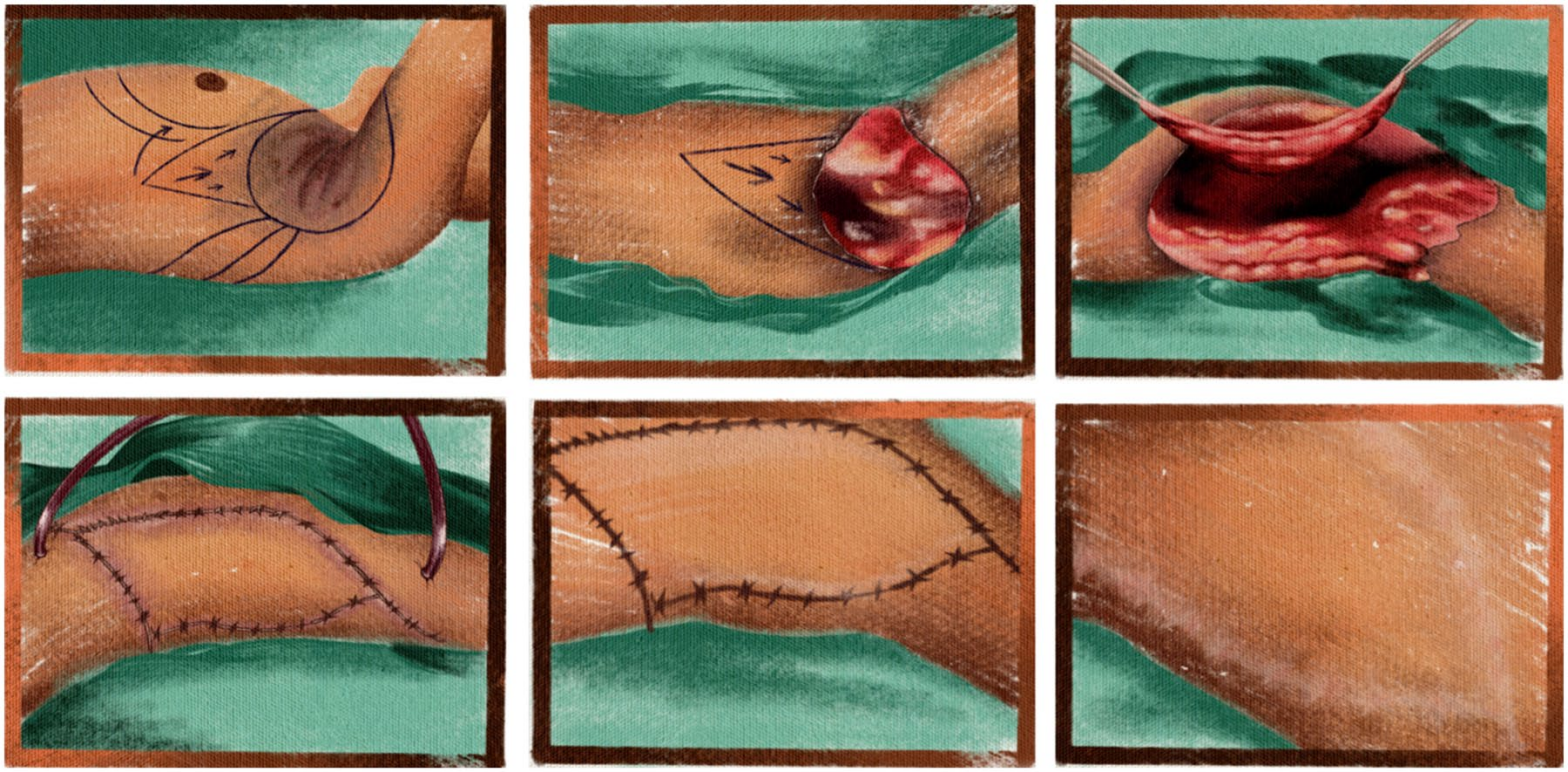
Thoracodorsal artery perforator–based V–Y advancement flap for axillary defect reconstruction. Preoperative markings outlining unilateral left axillary hidradenitis suppurativa and the planned V–Y advancement flap based on thoracodorsal artery perforators. Post-excision view demonstrating the surgical defect following wide excision. Intraoperative mobilization and advancement of the perforator flap for defect closure are shown. A closed-suction drain was placed at the donor site.

Primary closure is feasible when sufficient adjacent tissue laxity exists, often in less extensive disease. Split-thickness skin grafts are suitable for delayed reconstruction but carry risks of contracture, instability in high-mobility areas, and aesthetic mismatch. Local and regional flaps provide durable coverage, good tissue match, and reliable vascularity [13,14]. Thoracodorsal artery perforator (TDAP) flaps offer pliable coverage with preservation of shoulder function and primary donor-site closure [12,13] (Figure 5). When perforators are insufficient or unpredictable, a latissimus dorsi musculocutaneous flap provides a dependable alternative, supplying ample tissue and a robust vascular pedicle to facilitate rapid and safe defect coverage [12,13]. Random-pattern local flaps, parascapular flaps, and other perforator-based flaps have also shown favorable outcomes with low complication rates.

Evidence from the literature supports various reconstructive approaches. Virág et al. reported 22 HS-related defects treated with keystone perforator flaps, demonstrating high flap survival, minimal complications, and rapid functional recovery [14]. Similarly, Amendola et al. highlighted that regional axial flaps are associated with low complication and recurrence rates compared with skin grafts, supporting individualized, low-risk reconstruction for extensive HS [11].

In our series, latissimus dorsi musculocutaneous flaps were selected based on patient preference for shorter healing time and to facilitate early return to daily activities [15–17]. All patients had extensive axillary involvement with multiple tunnels, and preoperative Doppler evaluation identified variable perforator anatomy. Flap reconstruction allowed rapid obliteration of dead space, facilitated early shoulder mobilization, and resulted in satisfactory aesthetic and functional outcomes. No flap-related complications, donor-site morbidity, or recurrences were observed during follow-up.

These findings align with current recommendations emphasizing individualized surgical planning, the complementary role of medical therapy, and consideration of patient-centered outcomes. Both secondary intention healing and flap reconstruction are appropriate strategies for axillary HS, with the choice guided by patient priorities, wound characteristics, and institutional resources.

## Conclusion

Based on our experience, the systematic integration of wide excision with latissimus dorsi flap reconstruction provides durable coverage, preserves shoulder biomechanics, and supports optimal functional recovery. These findings underscore the need for individualized, low-risk reconstructive strategies in the surgical management of advanced HS and contribute to the growing evidence supporting the latissimus dorsi flap as a dependable workhorse in complex axillary reconstruction.
